# Association between insertion/deletion polymorphism in angiotensin-converting enzyme gene and acute lung injury/acute respiratory distress syndrome: a meta-analysis

**DOI:** 10.1186/1471-2350-13-76

**Published:** 2012-08-31

**Authors:** Akihisa Matsuda, Taro Kishi, Asha Jacob, Monowar Aziz, Ping Wang

**Affiliations:** 1 Department of Surgery, Hofstra North Shore-LIJ School of Medicine, 350 Community Drive, Manhasset, NY, 11030, USA; 2 Center for Immunology and Inflammation, The Feinstein Institute for Medical Research, 350 Community Drive, Manhasset, NY, 11030, USA; 3 Division of Psychiatry Research, The Zucker Hillside Hospital, Glen Oaks, NY, USA

**Keywords:** Angiotensin-converting enzyme (ACE) gene, Acute lung injury (ALI), Acute respiratory distress syndrome (ARDS), Meta-analysis

## Abstract

**Background:**

A previous meta-analysis reported a positive association between an insertion/deletion (I/D) polymorphism in the angiotensin-converting enzyme gene (*ACE*) and the risk of acute lung injury (ALI)/acute respiratory distress syndrome (ARDS). Here, we updated this meta-analysis and additionally assessed the association of this polymorphism with ALI/ARDS mortality.

**Methods:**

We searched electronic databases through October 2011 for the terms “angiotensin-converting enzyme gene”, “acute lung injury”, and “acute respiratory distress syndrome,” and reviewed all studies that reported the relationship of the I/D polymorphism in *ACE* with ALI/ARDS in humans. Seven studies met the inclusion criteria, comprising 532 ALI/ARDS patients, 3032 healthy controls, and 1432 patients without ALI/ARDS. We used three genetic models: the allele, dominant, and recessive models.

**Results:**

The *ACE* I/D polymorphism was not associated with susceptibility to ALI/ARDS for any genetic model. However, the *ACE* I/D polymorphism was associated with the mortality risk of ALI/ARDS in Asian subjects ( *P*_allele_ < 0.0001, *P*_dominant_ = 0.001, *P*_recessive_ = 0.002). This finding remained significant after correction for multiple comparisons.

**Conclusions:**

There is a possible association between the *ACE* I/D polymorphism genotype and the mortality risk of ALI/ARDS in Asians.

## Background

Acute lung injury (ALI) and its most severe form called acute respiratory distress syndrome (ARDS) are characterized by increased permeability of the alveolar-capillary barrier resulting in edema, excessive inflammatory responses, and interstitial fibrosis in the lung, thus impairing arterial oxygen exchange [1,2]. Despite innovations in intensive care medicine, the mortality of ARDS remains up to 40% [2]. Therefore, clarification of the unknown pathophysiology and the development of effective therapeutics are urgently needed to overcome the life-threatening conditions induced by ALI/ARDS. It has been suggested that the type and severity of injury may affect the occurrence of ALI/ARDS; however, it remains unclear why up to 50% of individuals are not affected despite experiencing similar injuries [3,4]. Several genetic variants have been suggested to be associated with the development and progression of ALI/ARDS (reviewed by Flores *et al*. [5]).

The human angiotensin-converting enzyme (ACE) gene (*ACE*) is located on chromosome 17q23 and contains an insertion/deletion (I/D) polymorphism of a 287-bpAlu repeat sequence in intron 16 [6,7]. Recently, strong evidence has accumulated for a pathophysiological association between lung and kidney diseases and the *ACE* I/D polymorphism, which is correlated with circulating and cellular ACE levels [8-12]. This evidence has been rendered using a meta-analysis approach that provided the most reliable compilation of the data currently available in the field [13,14]. The studies of Marshall *et al*. [15], Jerng *et al*. [16], Lu *et al*. [17], and Adamzik *et al*. [18] have shown a significant positive association between the *ACE* I/D polymorphism and the risk and/or mortality of ALI/ARDS, although the studies of Chan *et al*. [19], Plunkett *et al*. [20], and Villar *et al*. [21] failed to detect a positive association (Additional file 1: Table S1). This association therefore remains controversial. The discrepancy among these results may be because of different sample sizes, because the sample sizes of these studies were quite small for genetic association analysis. To overcome this limitation, Hu *et al*. [8] conducted a meta-analysis and reported that the *ACE* I/D polymorphism was associated with the risk of ALI/ARDS. However, we have noticed that two relevant studies were not included in this meta-analysis. Hence, to obtain a reliable conclusion regarding the association between the *ACE* I/D polymorphism and ALI/ARDS risk, we conducted an updated systematic review and meta-analysis of all relevant publications, increasing the sample size from 3133 (in Hu *et al.*[8]) to 4996. In addition, we carried out a meta-analysis between the *ACE* I/D polymorphism and the mortality of ALI/ARDS.

## Methods

We performed this meta-analysis according to the guidelines of Preferred Reporting Items for Systematic Reviews and Meta-analyses (PRISMA; 2009) [22].

### Data sources and searches

To identify eligible studies, we (A.M. and T.K.) independently searched PubMed, Embase, the Cochrane Library, and Google Scholar citations through October 2011 using as key words the terms “angiotensin-converting enzyme gene” or “ACE gene”; “acute lung injury” or “ALI”; and “acute respiratory distress syndrome” or “ARDS”.

### Study selection and eligibility criteria

We defined inclusion and exclusion criteria *a priori*. The inclusion criteria were: the study should (1) be published in a peer-reviewed journal, (2) contain independent data of *ACE* I/D genotype, (3) have the outcome as either development of ALI/ARDS or ALI/ARDS mortality, and (4) be a case–control study in human subjects. ALI/ARDS was defined as a lung disease with acute onset, non-cardiac diffuse bilateral pulmonary infiltrates, and a PaO_2_/FiO_2_ ≤ 300 for ALI and a PaO_2_/FiO_2_ ≤ 200 for ARDS [23]. Studies were excluded if they were duplicates. Studies included in the meta-analysis were not restricted by language.

### Data extraction

The reference lists of reviews and retrieved articles were verified independently by two investigators (A.M. and T.K.). The following information was extracted from each article: first author’s name, year of publication, ethnicity, patient group, age, sex, number of cases and controls, type and number of lung injury patients (ALI or ARDS) and controls for each *ACE* I/D genotype, and mortality at each assessment time. The frequencies of alleles were calculated for cases and controls from the corresponding genotype distributions. Disagreement was resolved by discussion.

### Statistical analysis

We conducted a meta-analysis between the *ACE* I/D polymorphism and the risk and mortality of ALI/ARDS. Because the mode of inheritance of ALI/ARDS is unknown, we performed a meta-analysis with the allele (I allele *versus* D allele), dominant (I/I *versus* I/D + D/D), and recessive (I/I + I/D *versus* D/D) models (as recommended in [24]). The meta-analysis was performed using Review Manager Version 5.0 for Windows (Cochrane Collaboration, http://www.cc-ims.net/RevMan). Cochran’s chi-square-based Q-statistic test was applied to assess between-study heterogeneity. We included healthy subjects and patients without ALI/ARDS (*e.g.*, intensive care unit patients who did not fulfill the criteria for ALI/ARDS) as separate controls. The pooled odds ratios (OR) were calculated using DerSimonian-Laird random-effects models [25] with 95% confidence intervals (CI) to measure the strength of the association. I^2^ indicates the percentage variance in the pooled OR that can be attributed to heterogeneity. Values of 25% are considered low, 50% moderate, and 75% high. We applied the conservative random effects model for all comparisons because the underlying effect may differ across studies and among heterogeneous populations. When we searched the HapMap database, we identified different linkage disequilibrium groups around the *ACE* gene among different ethnicities. Therefore, we conducted additional sensitivity analyses, separating the Caucasian and Asian participants. Finally, Bonferroni’s correction was used to control the type I error rate in the meta-analysis of mortality. We employed 18 separate tests (three genotype models: allele, dominant, and recessive; three populations: total, Caucasians, and Asians; and two traits: susceptibility and mortality); therefore, we calculated the corrected *P* value by multiplying the original *P* value by 18. *P* was considered significant at less than 0.05.

Publication bias was assessed using WINPEPI software (http://www.brixtonhealth.com/pepi4windows.html)[26], by visually examining a funnel plot and formally assessing asymmetry with both the Egger test [27] and the rank correlation test [28].

## Results

### Study characteristics

Our key word electronic database search yielded 119 references. According to the inclusion and exclusion criteria, we identified seven studies for meta-analysis of the association between *ACE* I/D polymorphism and ALI/ARDS [15-21]. All the studies were published in English. Four studies were conducted in Caucasians [15,18,20,21] and the remaining three studies were conducted in Asians [16,17,19]. All seven studies evaluated susceptibility to ALI/ARDS; four also evaluated the mortality of ALI/ARDS. The characteristics of the included studies are shown in Table 1.

**Table 1:** Information on the studies included in the meta-analysis

**Author**	**Year**	**Ethnicity**	**N**			**Age, yrs ± SD or (range)**			**Male sex, n (%)**			**Information**			**Mortality timing**
			**ALI/ARDS**	**Healthy controls**	**Controls**	**ALI/ARDS**	**Healthy controls**	**Controls**	**ALI/ARDS**	**Healthy controls**	**Controls**	**Diagnosis**	**Healthy controls**	**Controls**	
**Marshall**	2002	Caucasian	96	1906	262	50.3 (17–91)	N.A.	53.6	61 (63.5)	N.A.	162 (64.1)	ARDS	Healthy british men	“ICU control”; admitted to ICU for non-ARDS respiratory failure with mechanical ventilation (n=88). “CABG control”; admitted to ICU following CABG, but without respiratory failure (n=174).	-
**Chan**	2005	Chinese	17	326	123	59.1 (24–83)	42.5	36.9 (21–76)	9 (52.9)	172 (52.8%)	58 (47.2)	ARDS	Healthy individuals under routine health check	SARS patients without ARDS	-
**Jerng**	2006	Chinese	101	-	348	60±21	-	62.5	68 (68)	-	232 (66.7)	ARDS	-	“At-risk”; admitted to ICU with acute respiratory failure but not ARDS (n=138). “Non-at-risk”; no history of respiratory failure or hospitalization within 6M (n=210).	28 day
**Adamzik**	2007	German	84	200	-	43±16	N.A.	-	43 (51.2)	N.A.	-	ARDS	Healthy Caucasian individuals of either sex	-	30 day
**Villar**	2008	Spanish	120	364	92	66 (52–75)	(18–75)	71 (58–76)	76 (63.2)	N.A.	50 (54.3)	ARDS	population-based controls	Severe septic patients without ARDS but had some degree of repiartory failure.	ICU stay
**Plunket**	2008	Caucasian	13	-	199	7M (1-55M)	-	N.A.	7 (53.8)	-	N.A.	ARDS	-	Admitted to ICU for ventilatory and/or inotropic support without ARDS (n=199)	-
**Lu**	2011	Chinese	101	236	408	65.1±16.5	N.A.	N.A.	68 (67.3)	N.A.	N.A.	ALI	Healthy blood donors	Admitted to the hospital over the same period due to other diseases (n=408).	28 day
**Number of pooled studies in each phenotype**			**532**	**3032**	**1432**										

N.A.: not applicable.

### *ACE* I/D polymorphism and susceptibility to ALI/ARDS

#### ALI/ARDS patients *versus* healthy control subjects

Initially, we conducted a meta-analysis of ALI/ARDS risk using healthy control subjects, because this approach is more powerful than using other controls to confirm the association of genotype with disease susceptibility. Two studies [16,20] did not include healthy subjects as control in their analysis; therefore, we used the remaining five [15,17-19,21], comprising 418 ALI/ARDS patients and 3032 healthy control subjects. We found significant heterogeneities among the ORs (*P*_allele_ *<* 0.0001, *P*_dominant_ = 0.003, and *P*_recessive_ = 0.004) (Additional file 2: Figure S1, Additional file 3: Figure S2, Additional file 4: Figure S3). The pooled OR derived from the five studies [15,17,19,21,29] did not indicate significant association for any genotype model (Additional file 2: Figure S1, Additional file 3: Figure S2, Additional file 4: Figure S3). There was no significant publication bias using either the Egger test or the rank correlation test (data not shown). To limit the ethnic heterogeneity, we separated the studies into those with either Caucasian or Asian samples. In the three studies with Caucasian subjects [15,18,21], comprising 300 patients and 2470 healthy controls, we found significant heterogeneity among the ORs (*P*_allele_ < 0.0001, *P*_dominant_ = 0.001, *P*_recessive_ = 0.0006) (Additional file 2: Figure S1, Additional file 3: Figure S2, Additional file 4: Figure S3). The pooled OR indicated no significant association (Additional file 2: Figure S1, Additional file 3: Figure S2, Additional file 4: Figure S3). In the two studies with Asian subjects [17,19], comprising 118 patients and 562 healthy controls, there was no significant heterogeneity among the ORs (Additional file 2: Figure S1, Additional file 3: Figure S2, Additional file 4: Figure S3). The I/D polymorphism in *ACE* was not associated with susceptibility to ALI/ARDS in Asian subjects (Additional file 2: Figure S1, Additional file 3: Figure S2, Additional file 4: Figure S3).

#### ALI/ARDS patients *versus* patients without ALI/ARDS

One study [18] did not include patients without ALI/ARDS as a control in the analysis; therefore, we used the remaining six [15-17,19-21], comprising 448 ALI/ARDS patients and 1432 patients without ALI/ARDS. There were significant heterogeneities among the ORs (*P*_allele_ *=* 0.0006, *P*_dominant_ *=* 0.004, and *P*_recessive_ = 0.03) (Additional file 5: Figure S4, Additional file 6: Figure S5, Additional file 7: Figure S6). The pooled OR derived from the six studies [15-17,19-21] did not indicate significant association for any genotype model (Additional file 5: Figure S4, Additional file 6: Figure S5, Additional file 7: Figure S6). There was no significant publication bias using either the Egger test or the rank correlation test (data not shown). We then conducted an additional analysis to limit the ethnic heterogeneity. In the three studies with Caucasian subjects [15,20,21], comprising 229 ALI/ARDS patients and 553 patients without ALI/ARDS, we found significant heterogeneity among the ORs (*P*_allele_ = 0.0009, *P*_dominant_ = 0.03, and *P*_recessive_ = 0.002) (Additional file 5: Figure S4, Additional file 6: Figure S5, Additional file 7: Figure S6). The pooled OR derived from the three studies [15,20,21] indicated no significant association for any genotype model (Additional file 5: Figure S4, Additional file 6: Figure S5, Additional file 7: Figure S6). In the three studies with Asian subjects [16,17,19], comprising 219 ALI/ARDS patients and 879 patients without ALI/ARDS, the I/D polymorphism in *ACE* was not associated with ALI/ARDS risk. There was no evidence of between-study heterogeneity (Additional file 5: Figure S4, Additional file 6: Figure S5, Additional file 7: Figure S6).

#### *ACE* I/D polymorphism and mortality of ALI/ARDS

**Figure 1:**
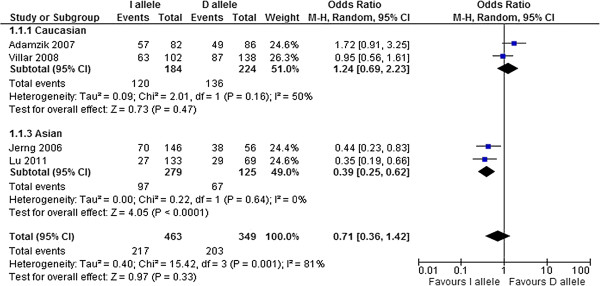
**Forest plot of OR with 95% CI for*ACE*I/D polymorphism in ALI/ARDS mortality: allele model.**

**Figure 2:**
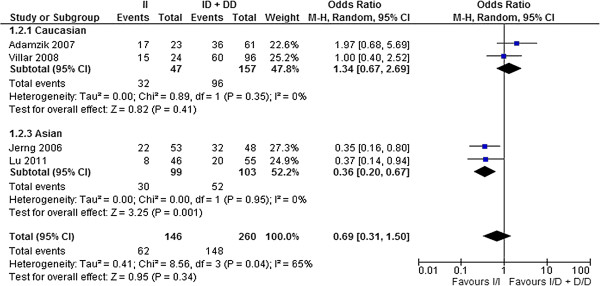
**Forest plot of OR with 95% CI for*ACE*I/D polymorphism in ALI/ARDS mortality: dominant model.**

**Figure 3:**
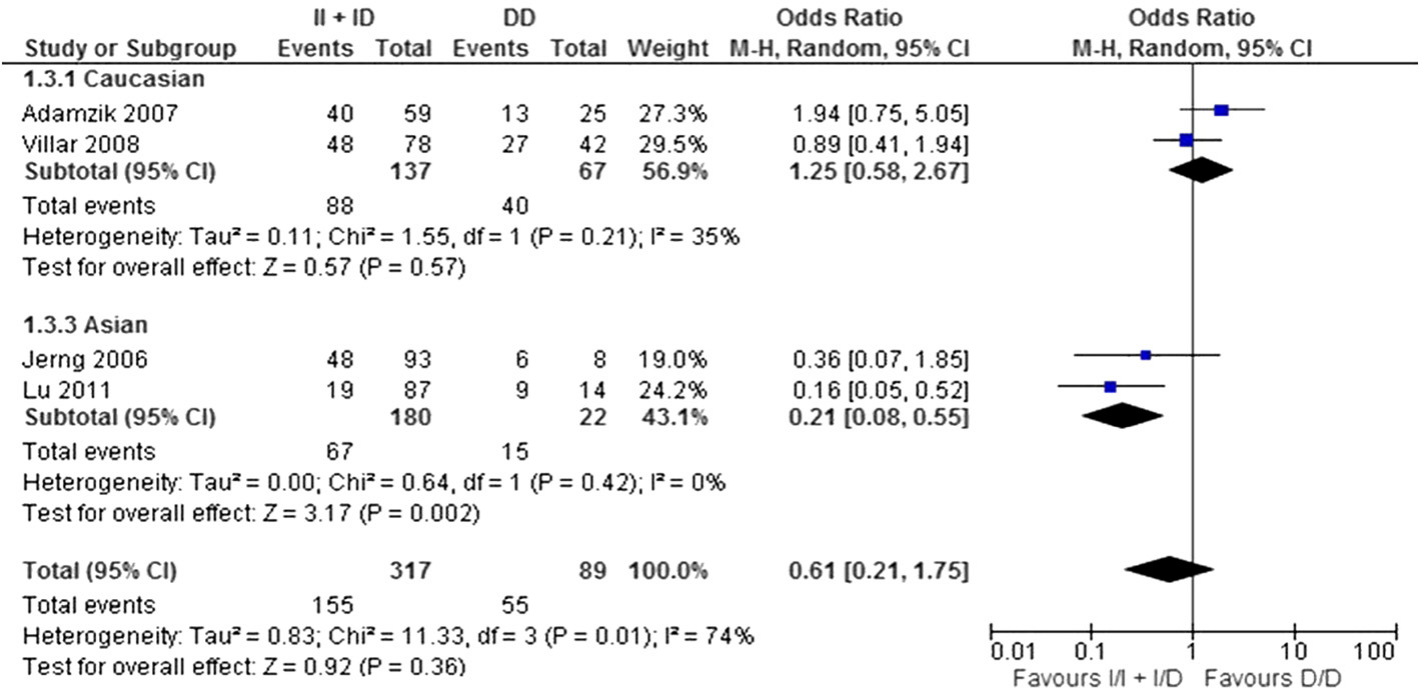
**Forest plot of OR with 95% CI for*ACE*I/D polymorphism in ALI/ARDS mortality: recessive model.**

In our meta-analysis, we used the four studies [16-18,21] that reported ALI/ARDS-related mortality, comprising 196 survivors and 210 non-survivors. We found significant heterogeneities among the ORs (*P*_allele_ = 0.001, *P*_dominant_ = 0.04 and *P*_recessive_ = 0.01) (Figures 1,2,3). The pooled OR derived from the four studies [16-18,21] did not indicate a significant association for any genotype model (Figures 1,2,3). There was no significant publication bias using either the Egger test or the rank correlation test (data not shown). In the two studies with Caucasian subjects [18,21], comprising 76 survivors and 128 non-survivors, we did not detect significant heterogeneity among the ORs (Figures 1,2,3). The pooled OR from the two studies [18,21] did not show a significant association for any genetic model (Figures 1,2,3). In the two studies with Asian subjects [16,17], comprising 120 survivors and 82 non-survivors, we did not detect significant heterogeneity among the ORs (Figures 1,2,3). The pooled OR from the two studies [16,17] did indicate a significant association for all genetic models (*P*_allele_ < 0.0001, *P*_dominant_ = 0.001, *P*_recessive_ = 0.002) (Figures 1,2and3). Moreover, the pooled OR remained significant after Bonferroni’s correction (corrected *P*_allele_ < 0.0001, corrected *P*_dominant_ = 0.018, corrected *P*_recessive_ = 0.036).

## Discussion

The renin-angiotensin system (RAS) has been well documented to contribute to the pathophysiology of ALI/ARDS by increasing vascular permeability [30]. ACE is a key enzyme of the RAS that converts inactive angiotensin I to the vasoactive and aldosterone-stimulating peptide angiotensin II that metabolizes kinins and many other biologically active peptides [31]. ACE is found in varying amounts on the surface of lung epithelial and endothelial cells. Activation of the RAS can stimulate production of tumor necrosis factor alpha in cardiac fibroblasts [32]. Angiotensin II induces apoptosis of lung epithelial and endothelial cells and is a potent fibrogenic factor [33-36]. Based on these biological properties of ACE, there is considerable interest in its potential involvement in ALI/ARDS.

Despite conflicting findings among the genetic association studies, a recent meta-analysis [8] suggested a positive association between the *ACE* I/D polymorphism and ARDS susceptibility among Caucasians. Here, we have extended this meta-analysis to a much larger sample size (532 ALI/ARDS patients, 3032 healthy controls, and 1432 patients without ALI/ARDS), and failed to detect an association between the *ACE* I/D polymorphism and ALI/ARDS risk. The discrepancy may be because of the different methodology used and the different sample size. We believe that our result is more convincing because we added two studies to those used by Hu *et al*. [8] and also used three genetic models, as recommended in the literature [24]. Furthermore, we extended our meta-analysis to examine mortality, and detected a significant association between the *ACE* I/D polymorphism and the mortality risk of ALI/ARDS in Asian populations.

ALI/ARDS patients had a higher frequency of the D/D genotype than controls did (Additional file 1: Table S1). The *ACE* I/D polymorphism has been associated with 28–47% of the variance in circulating ACE levels in healthy subjects, and high plasma ACE levels are associated with the D/D genotype [6,7]. It is therefore possible that the high plasma ACE levels produced by having the D/D genotype affect the mortality of ALI/ARDS. Recently, ACE inhibitors have been reported to attenuate ALI induced by bleomycin, acid, and endotoxin in animal models [37-39]. Taken together, patients at risk of mortality from ALI/ARDS, as determined by *ACE* I/D polymorphism screening, may be good candidates for treatment with ACE inhibitors. Furthermore, because our results suggest that the *ACE* I/D polymorphism genotype might influence treatment outcome through differences in drug metabolism and activity, it will be important to investigate the pharmacogenomics of ALI/ARDS and gene–gene interactions in the RAS to clarify the role of different RAS genes in the pathophysiology and treatment response of ALI/ARDS.

We did not find an association between the *ACE* I/D polymorphism and ALI/ARDS mortality in the total population. However, there were significant heterogeneities among the ORs. This heterogeneity may be derived from: (1) different ancestries, (2) incomplete genotyping or genotyping error differences among the studies, and (3) a relatively small total sample size (196 survivors and 210 non-survivors). Differences in the genotype distributions have been reported between Caucasians and Asians, which may support the above explanations (the studies of Villar *et al.*[21] and Adamzik *et al.*[18] reported that the major allele was “Deletion”, but other Asian studies showed that the major allele was “Insertion”). Therefore, we stratified the population into Caucasian and Asian subjects, thereby eliminating the significant heterogeneity among the ORs for both ethnicities. After stratification, we detected a significant association between the *ACE* I/D polymorphism and ALI/ARDS mortality in Asian populations. D/D was the risk genotype for mortality in Asian ALI/ARDS patients.

Several limitations of our meta-analysis remain, *e.g.*, a small sample size and differences in the quality control among the studies. Neither the mode of inheritance nor the heritability of ALI/ARDS is known. Therefore, we used three genetic models to obtain more statistical power than in previous individual studies. Based on the common disease-common variant hypothesis, and assuming ALI/ARDS to be a complex disease, at least 4000 samples ( *i.e.*, 2000 cases and 2000 controls) are required to obtain sufficient statistical power (GRR = 2.0 and *P* = 5 × 10^-7^) [40]. Given that our study is under-powered because of its small sample size, we may have failed to detect a genuine association between the *ACE* I/D polymorphism genotype and ALI/ARDS susceptibility. A replication study using a larger sample size, and/or samples from other populations, is required to obtain conclusive results.

## Conclusions

In conclusion, our results indicate that the genotype of the I/D polymorphism in *ACE* may be a predictor of ALI/ARDS mortality in Asian populations. However, more case-control association investigations on larger, stratified populations are required to clarify the role of this polymorphism in ALI/ARDS risk and mortality.

## Competing interests

The authors declare that they have no competing interests.

## Authors’ contributions

AM and TK had full access to all of the data in the study and take responsibility for the integrity of the data and the accuracy of the data analysis. AM and TK designed the study, and acquired and analyzed the data. AM and TK drafted the manuscript. MA and AJ helped prepare the manuscript. PW supervised the study. All authors have read and approved the final manuscript.

## Pre-publication history

The pre-publication history for this paper can be accessed here:

http://www.biomedcentral.com/1471-2350/13/76/prepub

## Supplementary Material

Additional file 1 **Table S1.** Genotype distribution, MAF, and GRR of the studies included in the meta-analysis. Click here for file

Additional file 2 **Figure S1.** Forest plot of OR with 95% CI for *ACE* I/D polymorphism in ALI/ARDS susceptibility: allele model. Control: healthy control subjects. Click here for file

Additional file 3 **Figure S2.** Forest plot of OR with 95% CI for *ACE* I/D polymorphism in ALI/ARDS susceptibility: dominant model. Control: healthy control subjects. Click here for file

Additional file 4 **Figure S3.** Forest plot of OR with 95% CI for *ACE* I/D polymorphism in ALI/ARDS susceptibility: recessive model. Control: healthy control subjects. Click here for file

Additional file 5 **Figure S4.** Forest plot of OR with 95% CI for *ACE* I/D polymorphism in ALI/ARDS susceptibility: allele model. Control: patients without ALI/ARDS. Click here for file

Additional file 6 **Figure S5.** Forest plot of OR with 95% CI for *ACE* I/D polymorphism in ALI/ARDS susceptibility: dominant model. Control: patients without ALI/ARDS. Click here for file

Additional file 7 **Figure 6.** Forest plot of OR with 95% CI for *ACE* I/D polymorphism in ALI/ARDS susceptibility: recessive model. Control: patients without ALI/ARDS. Click here for file
