# Supplementary figures and images for: Association between insertion/deletion polymorphism in angiotensin-converting enzyme gene and acute lung injury/acute respiratory distress syndrome: a meta-analysis
Source: BMC Med Genet. 2012 Aug 31;13:76. doi: 10.1186/1471-2350-13-76 (PMC3459791; doi:10.1186/1471-2350-13-76)

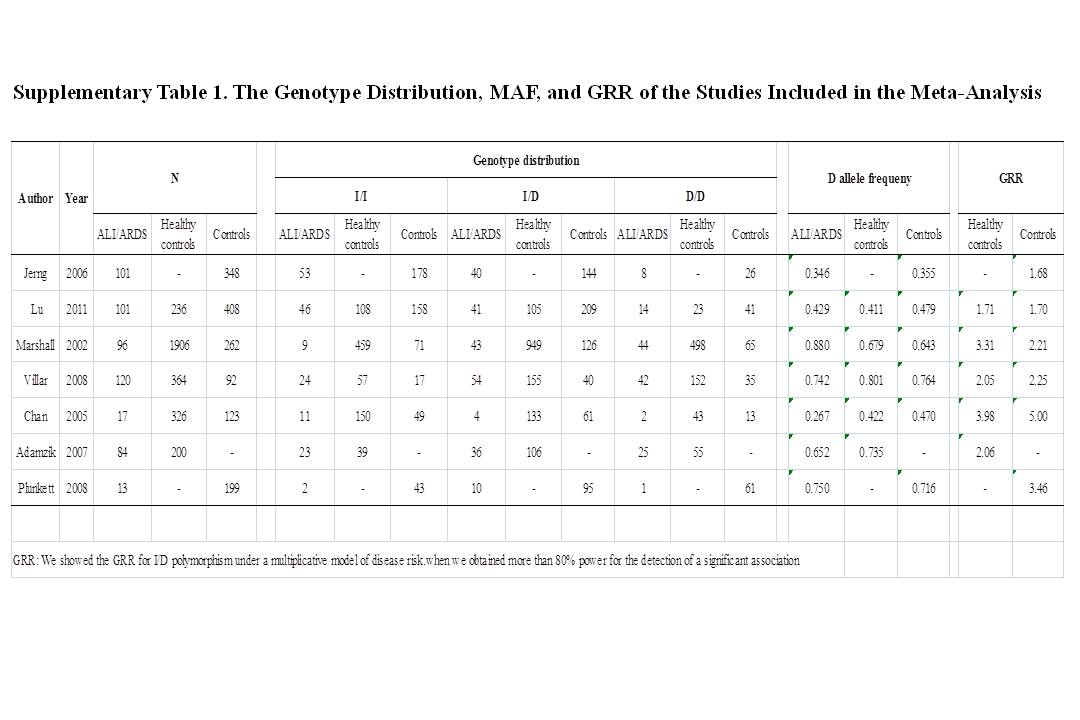

Supplement: Additional file 1 — Table S1. Genotype distribution, MAF, and GRR of the studies included in the meta-analysis. [file 1471-2350-13-76-S1.jpeg]

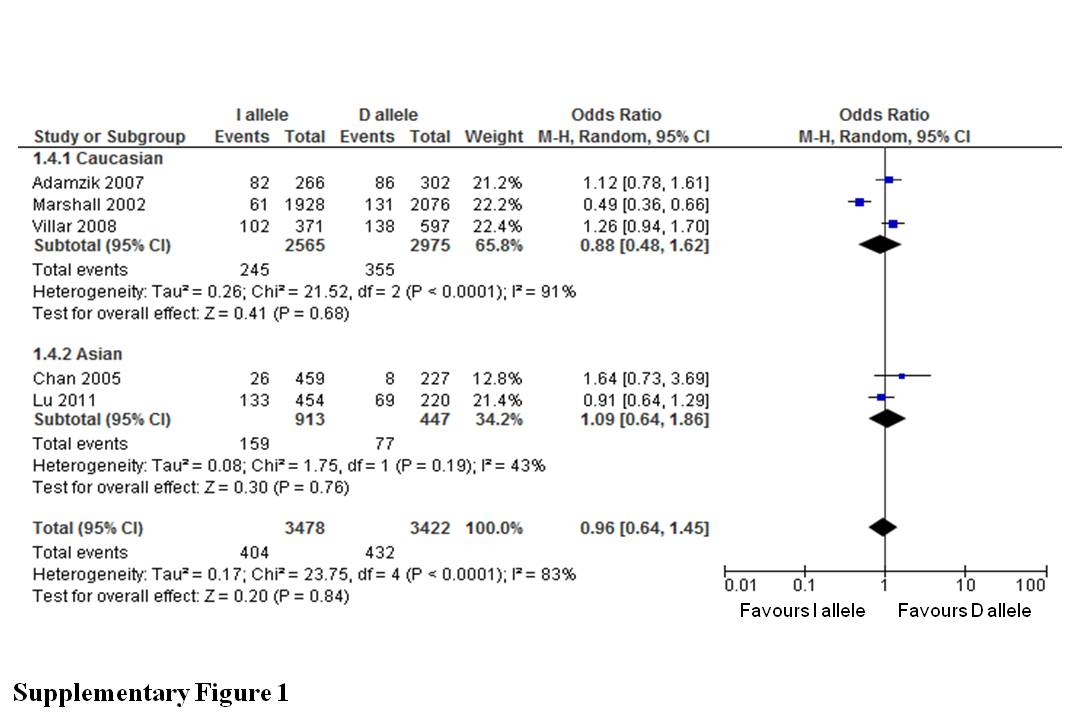

Supplement: Additional file 2 — Figure S1. Forest plot of OR with 95% CI for ACE I/D polymorphism in ALI/ARDS susceptibility: allele model. Control: healthy control subjects. [file 1471-2350-13-76-S2.jpeg]

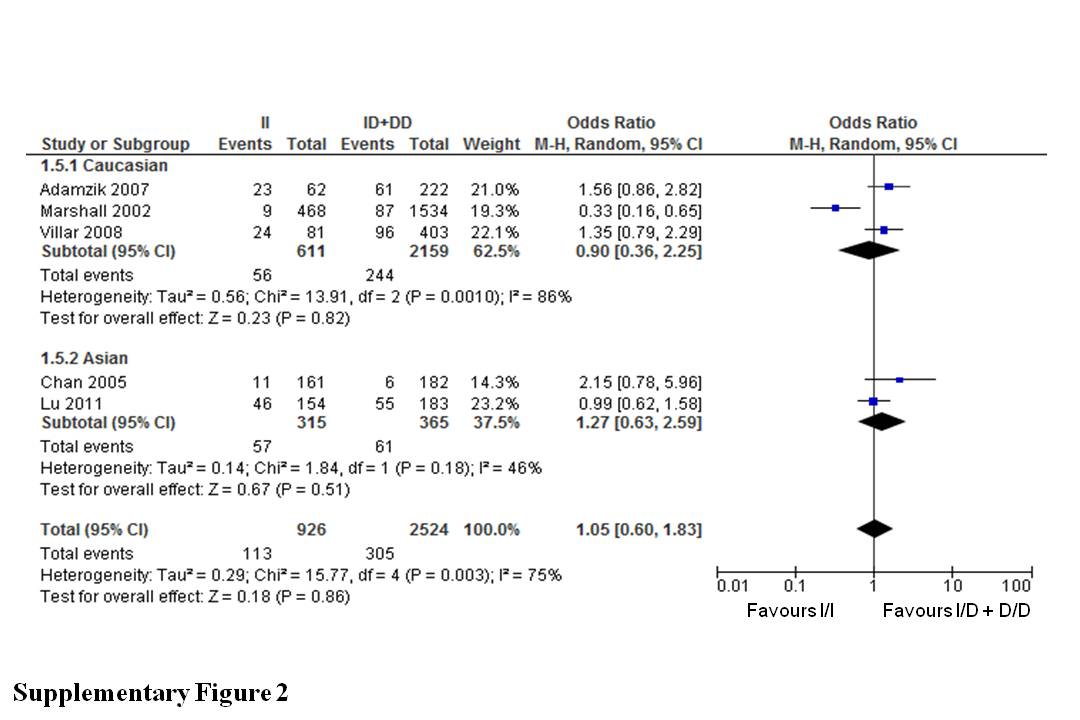

Supplement: Additional file 3 — Figure S2. Forest plot of OR with 95% CI for ACE I/D polymorphism in ALI/ARDS susceptibility: dominant model. Control: healthy control subjects. [file 1471-2350-13-76-S3.jpeg]

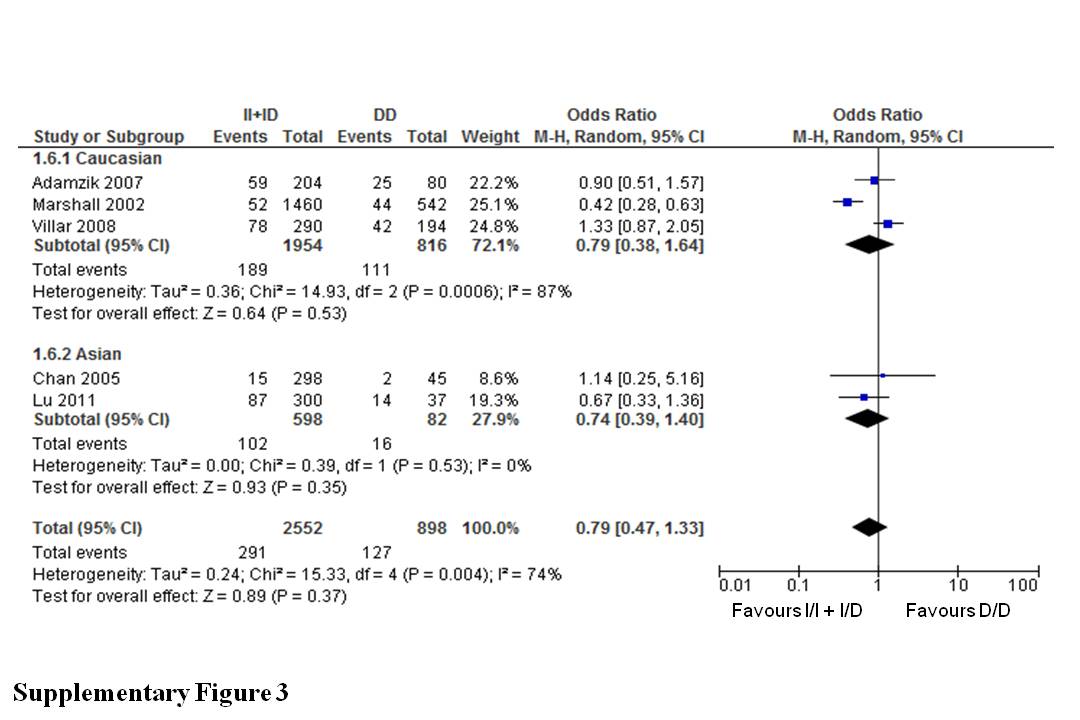

Supplement: Additional file 4 — Figure S3. Forest plot of OR with 95% CI for ACE I/D polymorphism in ALI/ARDS susceptibility: recessive model. Control: healthy control subjects. [file 1471-2350-13-76-S4.jpeg]

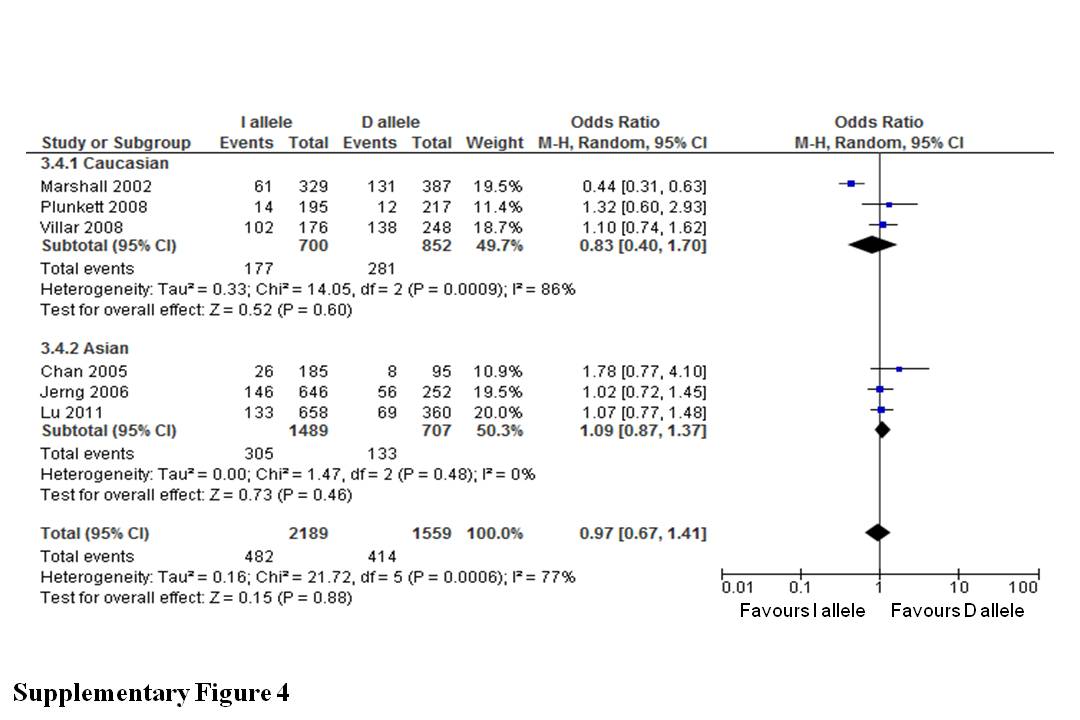

Supplement: Additional file 5 — Figure S4. Forest plot of OR with 95% CI for ACE I/D polymorphism in ALI/ARDS susceptibility: allele model. Control: patients without ALI/ARDS. [file 1471-2350-13-76-S5.jpeg]

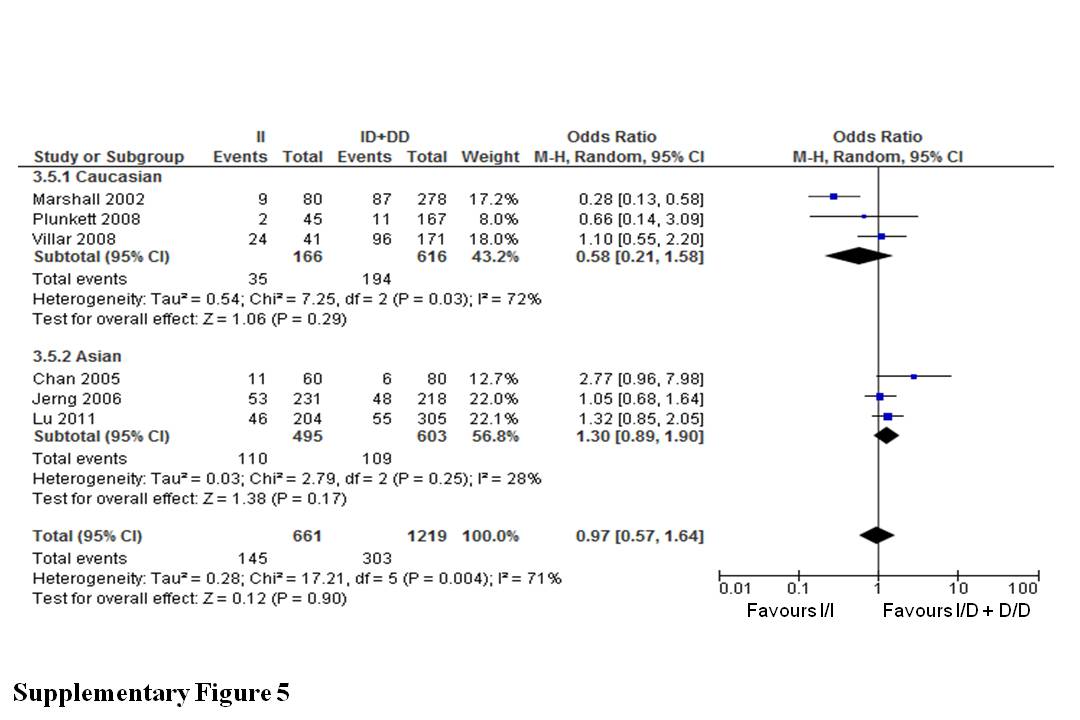

Supplement: Additional file 6 — Figure S5. Forest plot of OR with 95% CI for ACE I/D polymorphism in ALI/ARDS susceptibility: dominant model. Control: patients without ALI/ARDS. [file 1471-2350-13-76-S6.jpeg]

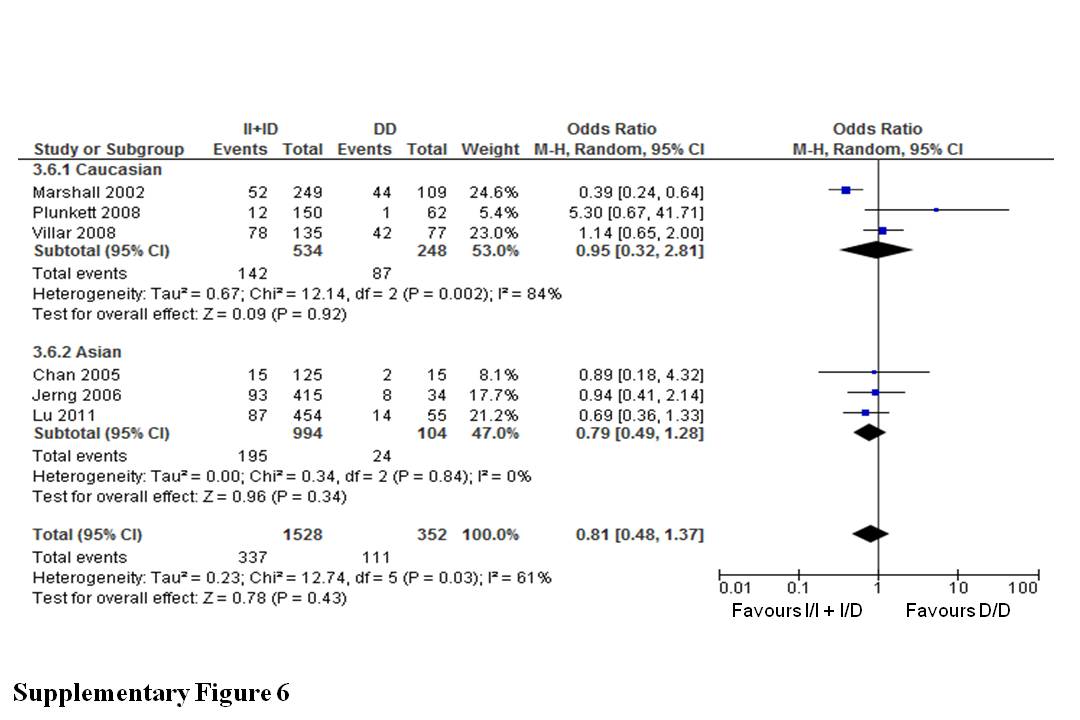

Supplement: Additional file 7 — Figure 6. Forest plot of OR with 95% CI for ACE I/D polymorphism in ALI/ARDS susceptibility: recessive model. Control: patients without ALI/ARDS. [file 1471-2350-13-76-S7.jpeg]
